# Long-term survival, temperature, and torpor patterns

**DOI:** 10.1038/s41598-023-33646-6

**Published:** 2023-04-24

**Authors:** Fritz Geiser, Thomas Ruf

**Affiliations:** 1 Centre for Behavioural and Physiological Ecology, Zoology, University of New England, Armidale, 2351 Australia; 2 Department of Interdisciplinary Life Sciences, Research Institute of Wildlife Ecology, University of Veterinary Medicine, Savoyenstrasse 1, 1160 Vienna, Austria

**Keywords:** Ecology, Evolution, Physiology, Zoology

## Abstract

Mammalian and avian torpor is highly effective in reducing energy expenditure. However, the extent of energy savings achieved and thus long-term survival appear to differ between species capable of multiday hibernation and species restricted to daily heterothermy, which could, however, be due to thermal effects. We tested how long-term survival on stored body fat (i.e. time to lean body mass), crucial for overcoming adverse periods, is related to the pattern of torpor expressed under different ambient temperatures (T_a_: 7 °C typical of hibernation, 15 and 22 °C typical of daily torpor) in the small marsupial hibernator the pygmy-possum (*Cercartetus nanus*). Possums expressed torpor at all T_a_s and survived without food for 310 days on average at T_a_ 7 °C, 195 days at T_a_ 15 °C, and 127 days at T_a_ 22 °C. At T_a_ 7 and 15 °C, torpor bout duration (TBD) increased from < 1–3 to ~ 5–16 days over 2 months, whereas at T_a_ 22 °C, TBD remained at < 1 to ~ 2 days. At all T_a_s daily energy use was substantially lower and TBD and survival times of possums much longer (3–12 months) than in daily heterotherms (~ 10 days). Such pronounced differences in torpor patterns and survival times even under similar thermal conditions provide strong support for the concept that torpor in hibernators and daily heterotherms are physiologically distinct and have evolved for different ecological purposes.

## Introduction

Daily torpor and multiday torpor (hibernation) in mammals and birds are crucial survival strategies because they can substantially reduce the high energy expenditure associated with endothermy. Both are employed by many species under a variety of conditions but they are either viewed as (1) two physiologically distinct patterns of torpor characteristic for certain species, selected for different ecological purposes and represented by clearly different physiological variables, or (2) as a continuum of functional variables of a single but temperature-dependent torpor pattern^1^.

Mammalian hibernation or multiday torpor is characterized by pronounced reductions of body temperatures (T_b_) and energy expenditure^2–4^. The hibernation season, often from autumn to spring, typically is composed of a series of torpor bouts with a low T_b_ (~ 5 °C) and a torpor bout duration (TBD) of several days or weeks, which are interrupted by periodic, brief rewarming and normothermic periods with a high T_b_ (~ 37 °C) lasting for several hours^3,5,6^. Importantly and complicating terminology, although hibernators are capable of multiday torpor bouts, they also can express brief bouts of torpor lasting less than 1 day and these are typically observed at the beginning of the hibernation season, at high T_a_, or when the animal is exposed to a fluctuating T_a_^7–11^. Metabolic rates during torpor (TMR) in small hibernators can be reduced to as little as ~ 1–2% of the basal metabolic rate (BMR), whereas the energy expenditure during the entire hibernation season is usually ~ 5–20% of that in active individuals because of the energetically expensive periodic arousals that consume most of the energy during the hibernation season^12–14^. Nevertheless, many hibernators can survive for months relying entirely on stored body fat accumulated before the hibernation season^3,12,15,16^ and many species in cold climates show little or no foraging during the hibernation season.

In contrast, in mammalian daily heterotherms, which use daily torpor exclusively and seem incapable of expressing multiday torpor, TBD is only 0.34 days on average^6^. Daily heterotherms typically forage on a daily basis including during the torpor season, many show little or no fattening and some species, to reduce overall energy expenditure, even may lose body mass before the season they express torpor most regularly (e.g.^17,18^). The T_b_ during daily torpor is on average reduced to ~ 18 °C, the TMR to ~ 30% of BMR, much higher than in hibernators^6^, and the heart rates in torpid daily heterotherms are much higher than those of hibernators even at the same T_b_^1^.

When functional variables of torpor or more specifically the physiologically possible capabilities of species are compared between the two groups of heterotherms, the mean and maximum TBD and the minimum TMR show strong bimodal distributions and differ significantly between the two groups^6^. The minimum T_b_s also differ significantly, but show some overlap^6^. However, an important point to consider in this context is that variables of torpor are strongly temperature-dependent und affected by the T_a_ the animal is exposed to. Especially exposure to a high T_a_ is not likely to reveal the physiological capability of a species in the cold. This is most easily seen for the T_b_, which in torpid animals follows the T_a_ often over a wide temperature range in both hibernators and daily heterotherms^9,19^ and because T_b_ approaches T_a_ but remains above T_a_ in both this can mask physiological differences. Moreover, during torpor entry and arousal from torpor the thermal response of T_b_ is to a large extent determined by the body mass of the animal and the prevailing T_a_ rather than the pattern of torpor expressed^20–23^. Nevertheless, it has been suggested that when the heterothermy index (HI) and other indices of heterotherms, based on such T_b_ measurements and quantified under different thermal conditions are compared, the two groups form a continuous single group with extreme values at either end^24^. This interpretation suggests there is only one functional group of heterothermic endotherms the physiology of which is simply a consequence of temperature and that the traditional classification of heterothermic mammals as hibernators and daily heterotherms in contrast to the homeotherms is clouded and possibly misleading^24^.

To resolve this controversy, crucial to the understanding of functional traits that have been selected to maximise survival in the wild, we quantified how TBD and loss of body mass of a small hibernator, reflecting energy expenditure and consequently long-term survival on stored body fat, are affected by T_a_. We tested these variables under thermal conditions that are similar to the minimum T_b_ of hibernators (T_a_ 7 °C) in comparison to mild thermal conditions (T_a_ 15 and 22 °C) approximating the minimum T_b_s of daily heterotherms. As long-term survival without food is one of the key adaptations of many hibernators, which have to survive on fat for months at low T_a_s in winter, this trait seems ultimately suited for tackling the question of whether there is one or two physiologically distinct groups of heterotherms.

We tested two hypotheses:

### Hypothesis 1

Survival to lean body mass, TBD and body mass loss of a small hibernator are similar to those of daily heterotherms at least at mild T_a_s. If this is the case the interpretation of a single torpor pattern with a temperature-dependent continuum of functional variables rather than different patterns of torpor is supported.

### Hypothesis 2

Survival to lean body mass, TBD and body mass loss of a small hibernator differ substantially from those of daily heterotherms including at mild T_a_s. If this is the case, the classification into two functionally different torpor patterns seems justified.

The species used for the experimental part of the study was the eastern pygmy-possum (*Cercartetus nanus*), referred to ‘possums’ in the text, a hibernator shown previously to be able express torpor under a variety of thermal conditions^25^ and with a lean body mass (~ 22 g) similar to that of the median (~ 26 g) of mammalian daily heterotherms^6^. This hibernating possum is well suited for the study because they fatten substantially and at high T_a_ they may express brief torpor bouts of < 1 day, temporally similar to the pattern in daily heterotherms. Consequently if functionally similar especially with regard to energy expenditure, this should be reflected in similar survival times. However, at low T_a_ they can remain torpid for up to 35 days^26^. The data on possums were compared with those of hibernators and daily heterotherms from the literature.

## Results

Possums maintained under natural autumn photoperiod, at T_a_ 20 °C and ad libitum food fattened extensively (Table 1). In all experimental groups, body mass reached around 2.5-fold the lean body mass, and the mean body masses when the torpor experiments began at the three experimental T_a_s of 7, 15, and 22 °C and food was withheld did not differ (*p* = 0.65) among the experimental groups (range of means: 53.8–56.6 g). The body mass when most stored fat was depleted at a lean body mass of ~ 22 g and torpor experiments were terminated was also similar (*p* = 0.13) among the experimental groups (range of means: 20.1–22.7 g). Consequently, the loss of body mass (range of means: 32.0–34.5 g) also was similar among the groups (*p* = 0.89). However, the loss of body mass per day without access to food differed significantly (*p* < 0.0001) among the experimental groups (range of means 0.107–0.27 g/day) and increased by ~ 1.7-fold from T_a_ 7–15 °C and by ~ 1.5-fold from T_a_ 15–22 °C (Table 1). The loss of body mass per day after the gut was voided and when TBD was near stable was lower than that measured over the entire torpor period (range of means 0.051–0.18 g/day), but also differed significantly (*p* < 0.0001) among the experimental groups (Table 1); it increased by 2.5-fold from T_a_ 7–15 °C and by 1.4-fold from T_a_ 15–22 °C.

**Table 1:** Body masses of pygmy possums (*Cercartetus nanus*) before and after the period of food withdrawal and the loss of body mass during that time.

Ambient temperature (°C)	Body mass	Body mass	Body mass	Body mass	Body mass
	Start (g)	End (g)	Loss (g)	Loss all (g/day)	Loss after > 38 days (g/day)
7	53.8 (1.4)	20.1 (0.2)	32.9 (1.4)	0.107 (0.004)	0.051 (0.006)
15	56.6 (2.7)	22.1 (0.5)	34.5 (2.2)	0.183 (0.010)	0.131 (0.013)
22	56.4 (2.1)	22.7 (0.7)	33.7 (2.5)	0.271 (0.014)	0.180 (0.009)
ANOVA F_2,12_	0.44	2.41	0.89	49.31	32.81
	*p* = 0.65	*p* = 0.13	*p* = 0.89	*p* < 0.0001	*p* < 0.0001

Body mass loss after > 38 days when the gut content was voided and TBD was stable is also shown for calculation of energy expenditure. Values are means with SE (in brackets) for n = 5 individuals at each T_a_. *P* values for differences in body mass among experimental groups at the beginning and end of the experiment and loss of body mass are provided.

Daily energy expenditure, estimated from body mass loss when TBD of possums was steady, was 2.0 kJ/day or 2% of the field metabolic rate (FMR) of a 30-g marsupial mammal (83.7 kJ/day^27^) at T_a_ 7 °C, 5.1 kJ/day or 6.1% of FMR at T_a_ 15 °C, and 7.0 kJ/day or 8.4% of FMR at T_a_ 22 °C. The daily energy expenditure of possums at the different T_a_s was only ~ 7–28% of the average daily metabolic rate of a 30-g daily heterotherm (~ 30 kJ/day) using daily torpor.

**Figure 1:**
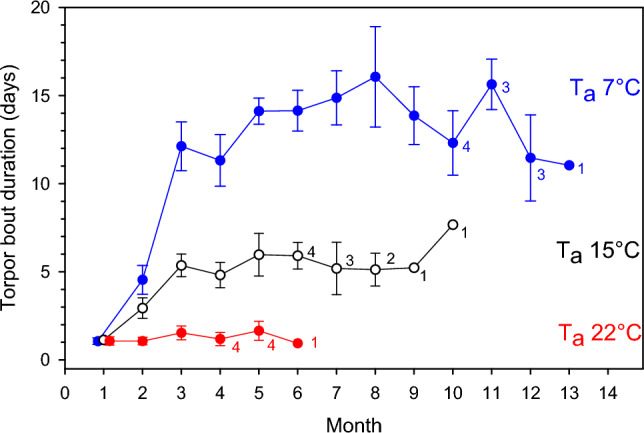
Torpor bout duration (TBD) as a function of time of pygmy-possums (*Cercartetus nanus*) hibernating at three different ambient temperatures (T_a_, 7 °C blue, 15 °C black and white, 22 °C red). Means with SE for n = 5 individuals at each T_a_ are shown, or numbers for individuals are shown next to the values. Both T_a_ (F = 69.1, *p* < 0.0001) and month (F = 37.0, *p* < 0.0001) had highly significant effects on TBD.

All possums expressed torpor at all T_a_s examined and both T_a_ (*p* < 0.0001) and month (*p* < 0.0001) had highly significant effects on TBD. Initially, animals rewarmed frequently and TBD was brief (< 1–3 days, Fig. 1). During the first month of torpor use, TBD was indistinguishable among thermal groups (1.1 ± 0.2 days at T_a_ 7 °C, 1.1 ± 0.2 days at T_a_ 15 °C; 1.1 ± 0.2 days at T_a_ 22 °C). The TBD at T_a_ 7 °C increased over the next two months to mean values ranging from 11.3 days to 16.1 days. All individuals at T_a_ 7 °C were able to hibernate on stored fat to month 9, four individuals to month 10, three individuals to month 12 and one individual to month 13. At T_a_ 15 °C, the TBD also increased over the first two months after food was withheld, but unlike at T_a_ 7 °C, the TBD increased only to mean values ranging from 4.8 to 6.0 days. All individuals at T_a_ 15 °C were able to hibernate on stored fat to month 5, four individuals to month 6, three individuals to month 7, two individuals to month 8 and one individual to month 10 with a mean TBD of 7.7 days in that month. At T_a_ 22 °C, TBD was similar to that of the other experimental groups on month 1 and lasted for 1.1 days. However, unlike for the other experimental groups, the TBD did not change substantially over the next months and mean TBDs at T_a_ 22 °C ranged between 1.1 and 1.6 days, but TBDs of < 1 day were observed in all months. Nevertheless, all individuals at T_a_ 22 °C were able to hibernate on stored fat to month 3, four individuals to month 5 and one individual to month 6 when its mean TBD was 0.9 days.

**Figure 2:**
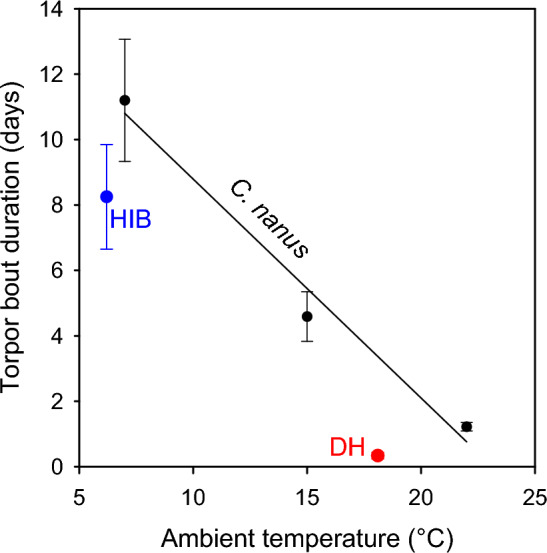
Mean torpor bout duration (TBD) of pygmy-possums (*Cercartetus nanus*) as a function of ambient temperature (T_a_). Mean TBD was strongly affected by T_a_: y = 15.48–0.67 x; r^2^ = 0.98. The mean TBD of other hibernators at the mean minimum T_b_ of hibernators (HIB blue dot, n = 70 species) and the mean TBD at the mean T_b_ of mammalian daily heterotherms (DH red dot, n = 50 species) are shown for comparison (data from^6^). The minimum T_b_ is shown on the T_a_ axis because the T_b_-T_a_ differential is small during steady-state torpor.

The mean TBD was strongly and negatively affected by T_a_ (Fig. 2). The mean TBD was 11.2 ± 1.9 days at T_a_ 7 °C, 4.6 ± 0.8 days at T_a_ 15 °C, and 1.2 ± 0.1 days at T_a_ 22 °C. TBD increased 9.3-fold from T_a_ 22 to 7 °C, 2.4-fold from T_a_ 15 to 7 °C, and 3.8-fold from T_a_ 22 to 15 °C. The mean TBD of possums at T_a_ 7 °C was slightly above that of mammalian hibernators (8.25 days, n = 70 species) at their mean T_b_ of 6.2 °C, but within the 95% confidence interval of hibernators^6^. In contrast at T_a_ 18.1 °C, equalling the mean minimum T_b_ of mammalian daily heterotherms, the TBD of possums was 9.9-fold of the mean TBD of daily heterotherms (0.34 days, n = 50 species) and well above the 95% confidence interval of daily heterotherms^6^.

**Figure 3:**
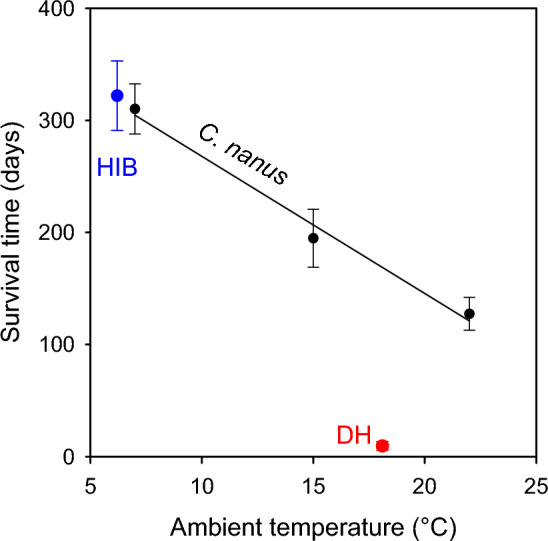
The survival times (time to lean body mass) without food of pygmy-possums (*Cercartetus nanus*) held at three ambient temperatures (T_a_). Means with SE for n = 5 individuals at each T_a_ are shown. Survival times differed significantly among the three groups (F_2,12_ = 18.52; *p* < 0.001). Mean survival times were a linear function of T_a_: y = 390.3–12.34 x; r^2^ = 0.99. The mean survival time without food of other hibernators (3 species at a mean body mass of 50 g^17^) at the mean minimum T_b_ of hibernators (HIB blue dot) and the mean survival time at the mean T_b_ of mammalian daily heterotherms (DH red dot) are shown for comparison (T_b_ data from^6^; survival times of daily heterotherms were for 4 species (*Apus apus* juvenile, *Sminthopsis crassicaudata*, *Dasycercus cristicauda*, *Peromyscus maniculatus bairdi*) at a mean body mass of 48 g from^33,44,45^). The minimum T_b_ is shown on the T_a_ axis because the T_b_ − T_a_ differential is small during steady-state torpor.

The mean time possums were able to survive on stored fat or the time to lean body mass differed significantly (*p* < 0.001) among the three experimental groups (Fig. 3). At T_a_ 7 °C, mean survival time was 310 days, 1.6-fold the survival time of possums at T_a_ 15 °C, where they survived on stored fat for 195 days on average. At T_a_ 22 °C, mean survival time was 127 days, 40% of that at T_a_ 7 °C and 65% of that at T_a_ 15 °C. The 9.3-fold increase in TBD from 1.2 days at T_a_ 22 °C to 11.2 days at T_a_ 7 °C was accompanied by a 2.43-fold increase in survival time. The mean survival time of three small hibernators (322 ± 31 days) at the mean minimum T_b_ of hibernators was similar to that of possums (Fig. 3). In contrast, the mean survival times of four small daily heterotherms (9.5 ± 3.9 days) at the mean minimum T_b_ of 18.1 °C was only 5.7% of possums at that temperature.

**Figure 4:**
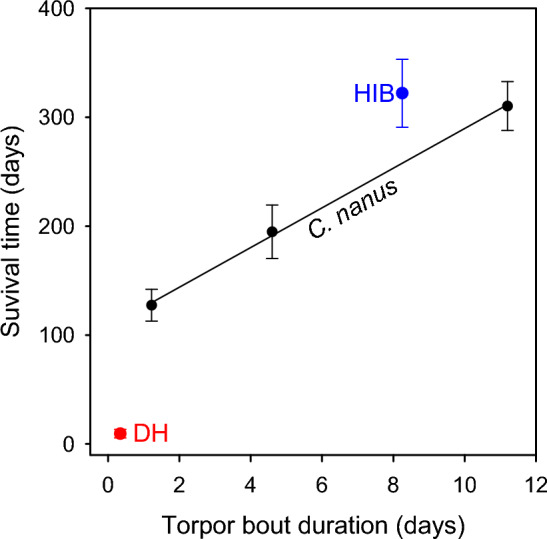
The survival times (time to lean body mass) without food of pygmy-possums (*Cercartetus nanus*) as a function of torpor bout duration (TBD). Mean survival times were a linear function of TBD: y = 107.5 + 18.2 x; r^2^ = 0.99. The mean survival times of small hibernators and daily heterotherms as in Fig. 3 at the mean TBDs for the groups from^6^.

Considering the relationships between T_a_ and TBD, and T_a_ and survival time, it is not surprising that TBD and mean survival times were strongly correlated (Fig. 4). A mean TBD of 1.2 days in possums was accompanied by a mean survival time of 127 days, whereas a mean TBD of 11.2 days by a mean survival time of 310 days. Thus a 1-day increase in TBD resulted in a 0.24-fold increase in possum survival time. While the survival times of small hibernators at the mean TBD was similar to that of possums, the survival times of daily heterotherms at the mean TBD was only 7% of that in possums.

## Discussion

Our study shows that irrespective of the thermal conditions, possums capable of hibernation, were able to survive for months on stored fat including when exposed thermal conditions experienced by daily heterotherms during daily torpor. The TBD of possums was strongly temperature-dependent, but even when exposed to a high T_a_, torpor bouts, similar to 13-lined ground squirrels^5^, were much longer than in daily heterotherms, resulting in comparatively lower mass loss, lower energy expenditure and much longer survival times in the small hibernator.

Thus, Hypothesis 1, which tested whether TBD, survival to lean body mass and mass loss of the small hibernator are similar to that of daily heterotherms at least at mild T_a_s, is not supported. Both torpor patterns and survival differed between possums capable of multiday hibernation and daily heterotherms even at high T_a_s when TBDs of possums were relatively short. In contrast, Hypothesis 2, which predicted that the small hibernator differs from daily heterotherms with regard to torpor patterns and survival times even at high T_a_s is supported by the observation of clearly distinct patterns of torpor pattern of possums and consequently much longer survival times than those expressed by daily heterotherms at all T_a_s investigated. This provides strong support for the view that daily torpor and hibernation are physiologically distinct. Rather than forming a continuum, the two patterns of heterothermy differ functionally from each other and also from the homeotherms. Thus, from a thermal energetics perspective, the three groups of endotherms, hibernators, daily heterotherms and homeotherms appear to represent ‘punctuated equilibria’.

With regard to the two groups of heterotherms, although torpor in both serves as energy and water conserving mechanism, the patterns appear to have been selected for different purposes. Hibernation in fat-storing hibernators aids primarily long-term survival of adverse conditions without or limited access to food^3^, whereas daily torpor assists in minimising daily energy expenditure and foraging requirements often under any environmental and trophic conditions and for adjustment of energy expenditure to energy availability^28^.

At all T_a_s examined, once possums had settled into a pattern of steady torpor bouts, which importantly was not instant, but took ~ 2 months at T_a_ 7 and 15 °C (Fig. 1), mass loss and consequently energy expenditure were very low. Although the values for daily energy expenditure observed here were affected by T_a_ and increased with T_a_, daily energy use of possums, despite periodic rewarming was a small fraction of the FMR of normothermic individuals, but similar to or at the low end of that observed in other hibernators^13,14^. Daily energy use of possums was however substantially lower (by ~ 70–90%) than in daily heterotherms expressing daily torpor under comparable thermal conditions.

Some of the differences observed for physiological variables between species expressing different patterns of torpor may raise the question as to why the interpretation of a single temperature-dependent pattern of torpor was developed. As outlined above this is largely related to the variables compared and their temperature-dependence. If T_b_ is compared it is important to consider that during torpor it is to some extent a function of T_a_ and, although T_b_ often may be a reasonable proxy for metabolic processes during torpor, it is the reduction of metabolism and water loss that are major purposes of torpor. Animals do not primarily enter torpor to reduce T_b_, but to lower energy and water use and odour towards predators^29^. Although a low T_b_ contributes to the reduction of these vital processes, its fall is unavoidable at T_a_s below the thermo-neutral zone because metabolism is reduced first at torpor entry and T_b_ typically follows.

Relying on T_b_ measurements to quantify torpor may be logistically the most feasible approach to quantifying temporal and thermal aspects of torpor in free-ranging animals, but its reliability as a proxy for physiological functions and especially energy expenditure is not as precise as is often assumed or implied. Energy savings are typically viewed to differ between hibernators and daily heterotherms because, in addition to longer TBDs, the former use metabolic inhibition more extensively and can reduce metabolism substantially with only small reductions in T_b_^26,30,31^. Differences in heart rates reflecting metabolic rate also differ substantially between hibernators and daily heterotherms even at the same T_b_^1^. Another potential problem of relying on T_b_ measures for estimating energy expenditure is passive rewarming from torpor which has been observed in many species. For the same increase of T_b_ when it is raised passively, metabolism changes only little, which is in sharp contrast to endothermic rewarming which requires an enormous increase in metabolism with substantial consequences for energy budgets (see e.g.^32^).

With regard to comparisons of long-term survival on stored body fat it is of course important to consider the substantial differences in fattening in hibernators and specifically possums in contrast to many daily heterotherms. Different fat stores could be used to argue that simply the extent of fat storage is responsible for the observed differences and reject our arguments. However, all possums at all T_a_s examined were able to survive a ~ 60% reduction in body mass over > 120 days without food, whereas obese deer mice (*Peromyscus maniculatus*), typical daily heterotherms of similar size to possums, died after a ~ 30–50% reduction in body mass, but within only 3–5 days of food withdrawal^33^. Although fat storage is important in this context, the combined effects of prolonged torpor bouts and extremely low TMRs in hibernators are the traits that are mainly responsible for the observed differences with daily heterotherms and permit the slow use of stored fat. Interestingly, TMR and TBD also appear to be functionally linked^34^ and in turn likely contribute to the exceptional longevity observed for many hibernators^35^. Considering that both survival and TBD are strongly affected by T_a_ in possums it may not be especially surprising that survival is strongly affected by TBD, what is surprising is how strong the relationship is (Fig. 4).

Possums are not exceptional. For example, small (~ 10-g) insectivorous bats exhibit both short and multiday bouts of torpor often within the same season. Nevertheless, their minimum TMRs during torpor bouts of < 1 day are similar to those of similar-sized hibernators^10,36–38^. Even much larger hibernating dormice (*Glis glis*, ~ 150 g), exhibit both long and short bouts of torpor^7,16,39^. During short bouts TMR of *G. glis* was as low as 0.05 mlO_2_/g/h similar to that predicted for the minimum TMR of hibernators and much lower than that of daily heterotherms. Generally, the minimum TMR in hibernators, including during short bouts of torpor^1,10,37,38^ is less than 20% of that during daily torpor^6^, and one would therefore expect that fat reserves last proportionally longer in hibernators. This also could be one of the reasons why on average hibernators are larger than daily heterotherms^6^ to permit increased fat stores^40^.

Our data raise a conundrum in terminology with regard to the use of the term ‘daily torpor’. If we consider torpor solely from a temporal point of view, a T_b_ reduction for less than 1 day could be called ‘daily torpor’ for all species, but this would not reflect the same physiological response in all. In small bats, discussed above, torpor bouts in summer often last less than 1 day and are often referred to ‘daily torpor’ in the literature, but as stated above it has been shown for several species that even though they may appear thermally and temporally similar, metabolically they differ substantially from daily heterotherm (e.g.^1,37^). Thus, energetic estimates based on temporal variations of T_b_ only will be erroneous because short bouts of torpor in hibernators differ metabolically from those in daily heterotherms. Consequently, using ‘daily torpor’ to describe torpor bout of < 1 day in hibernators seems imprecise at best and this term should be reserved to the daily heterotherms, whereas in hibernators it should be called short bouts of torpor or similar to acknowledge the functional differences.

So why should we care whether there are 1 or 2 patterns of torpor? We should for several reasons. If all species were part of the same physiological group and only T_b_ or temperature effects were responsible for an observed heterothermic continuum, one would expect all species to perform the same under the same thermal conditions. Clearly, as we have shown here, this is not the case. Since the primary goal of hibernators seems to be survival without food for prolonged periods, whereas in daily heterotherms it is the reduction of energy expenditure to reduce food requirements during periods that include regular foraging, different physiological and behavioural mechanisms are required. Such behavioural, ecological and physiological differences will profoundly affect seasonal survival in relation to temporal changes in food availability and thermal conditions. Further, they are also crucial in relation to climate change with a predicted increase in temperature and the resulting new functional challenges that will require appropriate responses depending on the physiological capabilities of a species.

## Methods

### Study species and procedures

Possums captured at ~ 800 m elevation on the cool-temperate New England Tablelands (30° 22′ S, 152° 45′ E) near Dorrigo, New South Wales, Australia, were maintained at a T_a_ of 20 °C under natural photoperiod. Animals were held individually in cages provided with wood shavings and bedding, and were offered a surplus of food consisting of a mixture of high protein baby cereal, honey and water with added multivitamins and minerals, nuts and apples.

In austral autumn when body mass was high (> 50 g, ~ 2.5-fold that of lean adult mass, Table 1), individually caged animals (n = 5 for each thermal condition) and supplied with wood shavings, were transferred to temperature-controlled cabinets at one of three T_a_s: 7.3 ± 0.3 °C (SD), 15.0 ± 0.3 °C, and 21.7 ± 0.8 °C, referred to in the text as: T_a_ 7, 15, and 22 °C. A T_a_ of 7 °C is slightly above the mean minimum T_b_ (6.2 °C) of hibernating mammals and above the minimum T_b_ of *C. nanus*^6^. T_a_ 15 °C is slightly below the minimum T_b_ (16.9 °C) of a 30-g mammalian daily heterotherm and T_a_ 22 °C is similar to the minimum T_b_ (21.8 °C) of a 30-g avian daily heterotherm^6^. The photoperiod was L10:D14, which approximates the shortest yearly photoperiod within the species' range in south-eastern Australia; artificial light was provided by a dim incandescent bulb (10W). Water was freely available and food was withheld.

Periodic arousals from torpor were quantified non-invasively by passive infrared detectors that determined when each animal rewarmed periodically and re-entered torpor. For this purpose, passive infrared detectors (Jaycar Electronics LA-5017), which monitored the temperature profile of the cage and animal surface over an angle of 90º, were placed on top of each cage; activity events and thus arousals and normothermic periods were summed over 30 min and stored on a data logger (Electronic Services Unit, University of New England). Arousals were confirmed by checking daily whether fine sawdust that had been placed on animals when they first entered torpor had been removed^12^. Possums were weighed (to the nearest 0.1 g) at regular intervals to determine the loss of body mass over time and were removed from the experiment and offered food when their body mass reached ~ 22 g (Table 1), the lean mass of adults.

### Animal ethics declaration

This study was conducted under a scientific license provided by the NSW Parks and Wildlife Authority (SL100791) under the guidelines of the Environment Protection and Biodiversity Act 1999. Animal Ethics approval was granted by the animal ethics committee of the University of New England. The study was carried out in compliance with the ARRIVE guidelines on animal research and the Australian Code for the Care and Use of Animals for Scientific Research.

### Analyses

We measured and analysed body mass before and after the experiment, overall body mass loss, as well as body mass loss after the initial weeks of hibernation. The body mass loss after the initial hibernation phase (i.e. after > 38 days) when gut content was voided and TBD was steady was assumed to be entirely the result of fat metabolism^2^ because animals had access to water and likely maintained body water content at a steady percentage^41,42^. The energy consumption during this time was assumed to be equivalent to 38.9 kJ/g of fat lost^43^. Mean monthly TBD was determined and when a torpor bout extended over two months it was assigned to the month in which the greater part of the bout occurred. Survival times were calculated from the day food was removed and the animals were transferred to the temperature cabinet to the day they had reached their lean body mass (Table 1). Some of the data at T_a_ 7 °C were used in a paper^12^ with a different emphasis to the present study and all data were re-analysed for the purpose of the present study.

Measured values of TBD in possums were compared with those of daily heterotherms (n = 50 species) and hibernators (n = 70 species) as reported^6^. Survival times of hibernators without food were averaged for three species with a mean body mass of 50 g on average (from^17^). Survival times of daily heterotherms without food were obtained for four species (*Apus apus*, *Sminthopsis crassicaudata*, *Dasycercus cristicauda* and *Peromyscus maniculatus*) weighing 48 g on average^33,44,45^. Importantly, the three hibernators survived the food restrictions whereas the daily heterotherms died (as reflected by the times these experiments were permitted) and although there are data on physiological variables on many heterothermic species, data on survival times without food are scarce.

Numerical values in the results are expressed as means ± SE for n = 5 individuals at each T_a_. Residuals from all statistical models were normally distributed, as confirmed by visual inspection (quantile–quantile plots). Differences in body mass among experimental groups at the beginning and end of the experiment and loss of body mass were compared using one-way ANOVAs. Repeated measurements of TBD were analysed with a mixed effects model using the intercept as a random effect (R package lme4^46^ followed by ANOVA using package car^47^). Least squares regressions were used for linear fits.

## Data Availability

The datasets generated and analyzed during the current study will be made available by request to F. Geiser (fgeiser@une.edu.au).
